# Brain perivascular macrophages: current understanding and future prospects

**DOI:** 10.1093/brain/awad304

**Published:** 2023-09-11

**Authors:** Wenjie Wen, Jinping Cheng, Yamei Tang

**Affiliations:** Department of Neurology, Sun Yat-sen Memorial Hospital, Sun Yat-sen University, Guangzhou 510120, China; Brain Research Center, Sun Yat-sen Memorial Hospital, Sun Yat-sen University, Guangzhou 510120, China; Nanhai Translational Innovation Center of Precision Immunology, Sun Yat-sen Memorial Hospital, Foshan 528200, China; Department of Neurology, Sun Yat-sen Memorial Hospital, Sun Yat-sen University, Guangzhou 510120, China; Brain Research Center, Sun Yat-sen Memorial Hospital, Sun Yat-sen University, Guangzhou 510120, China; Nanhai Translational Innovation Center of Precision Immunology, Sun Yat-sen Memorial Hospital, Foshan 528200, China; Department of Neurology, Sun Yat-sen Memorial Hospital, Sun Yat-sen University, Guangzhou 510120, China; Brain Research Center, Sun Yat-sen Memorial Hospital, Sun Yat-sen University, Guangzhou 510120, China; Nanhai Translational Innovation Center of Precision Immunology, Sun Yat-sen Memorial Hospital, Foshan 528200, China

**Keywords:** brain perivascular macrophages, central nervous system diseases, physiological, pathological, therapy

## Abstract

Brain perivascular macrophages are specialized populations of macrophages that reside in the space around cerebral vessels, such as penetrating arteries and venules. With the help of cutting-edge technologies, such as cell fate mapping and single-cell multi-omics, their multifaceted, pivotal roles in phagocytosis, antigen presentation, vascular integrity maintenance and metabolic regulation have more recently been further revealed under physiological conditions.

Accumulating evidence also implies that perivascular macrophages are involved in the pathogenesis of neurodegenerative disease, cerebrovascular dysfunction, autoimmune disease, traumatic brain injury and epilepsy. They can act in either protective or detrimental ways depending on the disease course and stage. However, the underlying mechanisms of perivascular macrophages remain largely unknown. Therefore, we highlight potential future directions in research on perivascular macrophages, including the utilization of genetic mice and novel therapeutic strategies that target these unique immune cells for neuroprotective purposes.

In conclusion, this review provides a comprehensive update on the current knowledge of brain perivascular macrophages, shedding light on their pivotal roles in central nervous system health and disease.

## Teaser

Perivascular macrophages exist at the interface between blood vessels and the brain parenchyma and may act as immunological gatekeepers. Wen *et al.* provide a comprehensive overview of these cells and their roles in CNS health and disease.

## Introduction

Accumulating evidence has emerged supporting the involvement of brain-resident immune cells in the regulation of crucial processes such as development, homeostasis and disease. Among them, microglia in the brain parenchyma have been extensively studied for their roles in contributing to the immune regulation of the CNS. However, little is known about the functions of CNS border-associated macrophages, such as perivascular, leptomeningeal, choroid plexus and dural macrophages [also known as barrier-associated macrophages (BAMs)].^1^ This review aims to explore the fundamental functions of perivascular macrophages (PVMs), which exist at the interface between blood vessels and the brain parenchyma and act as a safeguard for the brain. We will discuss further how these features are disturbed by multifarious pathological factors.

### Discovery and definition of perivascular macrophages

In 1927, Kubie^2^ showed that trypan blue injected into the subarachnoid space could move down into the perivascular space (PVS) and be taken up by elongated cells. These cells, initially called granular pericytes, were later considered to be fluorescent granular perithelial cells that cleared metabolic waste from the brain.^3^ Subsequently, Hickey and Kimura^4^ suggested these cells as bone marrow-derived perivascular microglia, which were ED2-positive and capable of presenting antigens to lymphocytes.

**Figure 1:**
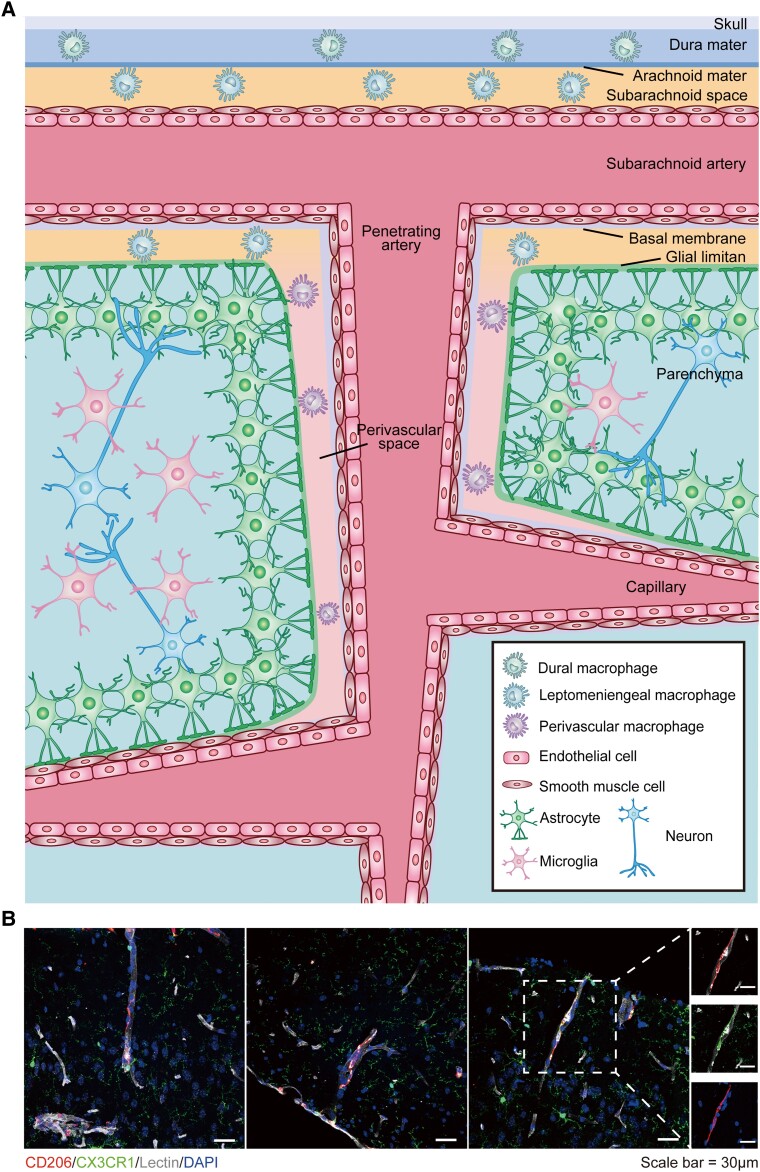
**Anatomic location of PVMs and other cells in the vicinity of perivascular space**. (**A**) PVMs are found in the fluid-filled perivascular space (PVS), also known as the Virchow-Robin space (VRS), which is enclosed by the vascular basement membrane and the glial limitans. In addition to PVMs, leptomeningeal macrophages are also found within the subarachnoid space. Dural macrophages reside in the dura mater, andeurons, astrocytes, microglia and oligodendrocytes are found in the parenchyma. (**B**) Anatomical location and morphology of the PVMs and leptomeningeal macrophages (red) in the vicinity of the vasculature (grey) were visualized by immunofluorescence in CX3CR1-GFP mice brain slices. These showed that PVMs were distributed in the perivascular space along the vessels with a diameter of ∼10–30 μm, while leptomeningeal macrophages were located in the subarachnoid space near the meninges. Some of the PVMs were observed to be double positive for CD206 and CX3CR1. PVM = perivascular macrophage.

It was later shown that these so-called ‘perivascular microglial cells’ are a distinct population of macrophages expressing CD163, which engulf substances in the PVS.^5–7^ The term ‘brain perivascular macrophage’ was coined to define them as a distinct population of cells located in the PVS surrounding arterioles and venules with a diameter of 10–35 μm (Fig. 1).^8^ It is worth noting that in an animal study, the formation of the PVS occurred around P3-P10 during the developmental stage, coinciding with the emergence of PVMs in that space (P7).^9^

With regard to morphology, PVMs have an elongated strip-like shape and distribute along the arterioles as visualized by immunofluorescent staining using CD206 antibodies.^10–13^

### Origin and turnover of perivascular macrophages

**Figure 2:**
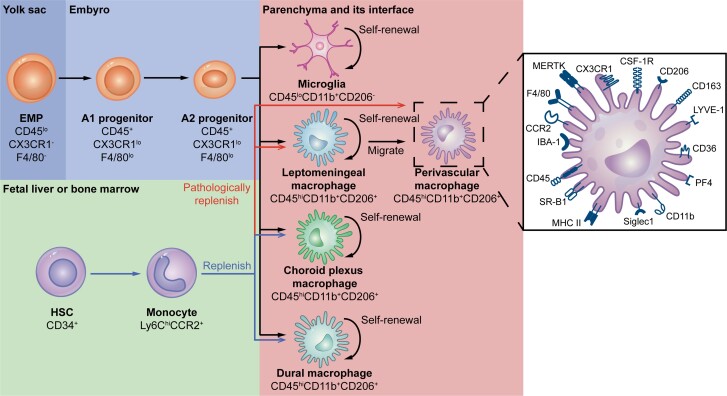
**Origin of PVMs and their characteristic markers**. Early erythromyeloid precursor (EMP) cells in the yolk sac differentiated into macrophage ancestor population A1 (CD45^+^CX3CR1^lo^F4/80^lo^) and progenitor population A2 (CD45^+^CX3CR1^hi^F4/80^hi^) in succession during embryonic development. The A2 progenitor then differentiated into CD206^−^ and CD206^+^ cells. The CD206^−^ cells converted into microglia, while the CD206^+^ cells transformed into BAMs, including PVMs, leptomeningeal macrophages, choroid plexus macrophages and dural macrophages. Surface markers of PVMs include molecules associated with development, phagocytosis, and antigen presentation, among others. Under physiological conditions, only choroid plexus macrophages and dural macrophages can be replenished by peripheral monocytes. Nevertheless, under pathological conditions, apart from the cells mentioned above, perivascular macrophages and leptomeningeal macrophages can also be supplemented by monocytes. PVM = perivascular macrophage.

It was first suggested that PVMs originate from the same source as other peripheral tissue-resident macrophages, including long-lived, yolk sac-derived and short-lived, bone marrow-derived macrophages.^14,15^ However, it has been recently reported that PVMs arose at E10.5 from early erythromyeloid precursor (EMP) cells located in the yolk sac.^1,16,17^ Following that, they became stable populations in the postnatal physiological state. It is worth noting that EMPs first differentiated into macrophage ancestor population A1 (CD45^+^CX3CR1^lo^F4/80^lo^) and progenitor population A2 (CD45^+^CX3CR1^hi^F4/80^hi^) during embryonic development. The A2 progenitor then differentiated into CD206^−^ and CD206^+^ cells with the expression of the transcription factors PU.1 and Irf8. The CD206^−^ cells converted into microglia, whereas the CD206^+^ cells transformed into BAMs, including PVMs (Fig. 2). In this process, the TGF-β signal and its downstream transcription factor SMAD family member 4 (SMAD4) were imperative for microglial development but dispensable for the development of PVMs.^17,18^ These findings indicate that PVMs have a pre-natal origin similar to that of microglia.

Nevertheless, according to a recent study by Masuda *et al*.,^9^ only leptomeningeal macrophages and microglia share a common prenatal progenitor, with PVMs arising from leptomeningeal macrophages postnatally in an integrin-dependent manner. Masuda *et al*.^9^ first investigated the distribution of CNS macrophages during embryogenesis using CX3CR1^GFP/+^ mice, finding that CX3CR1^+^CD206^−^ cells appeared in perivascular compartments at E12.5 and then disappeared before birth. Those cells, however, reappeared at P7 after birth. CX3CR1^+^CD206^+^ cells, on the other hand, first appeared in the meninges at E10.5, increased steadily and remained stable after birth. These results imply that there is a postnatal redistribution of PVMs.

To further distinguish the ontogeny of PVMs, Masuda *et al*.^9^ generated Mrc1^CreERT2^ mice. *Mrc1* is the coding gene for the protein CD206, which is explicitly expressed in BAMs rather than in microglia in the brain.^19^ Masuda *et al*.^9^ hypothesized that leptomeningeal macrophages could be a potential source of PVMs because these two cell types share common gene signatures.^20–22^ To confirm this hypothesis, they injected tamoxifen into pregnant Mrc1^CreERT2/CreERT2^; R26^tdT/tdT^ mice at E16.5 and found that almost all CD206^+^ leptomeningeal macrophages were labelled with tdTomato (tdT) fluorescent protein at P5 in the absence of tdT^+^ PVMs in the PVS. On P14, however, the percentage of tdT-expressing CD206^+^ PVMs was comparable to that of CD206^+^ leptomeningeal macrophages. Hence, they concluded that PVMs are generated postnatally, attributed to the infiltration of leptomeningeal macrophages. This has yet to be examined by an *in vivo* real-time experiment, such as with a two-photon microscope, which could be used to record the unfolding scene in the so-called peri-natal migration process. Moreover, with the aid of single-cell spatial transcriptomic analysis, the identities of the specific cell types of PVMs and leptomeningeal macrophages could be further revealed during a critical stage of the peri-natal period.

For a long time, it was thought that PVMs and leptomeningeal macrophages could be continuously replaced by bone marrow-derived cells.^4^ Recently, through the utilization of parabiosis and fate-mapping approaches in mice, Goldmann *et al*.^1^ found that the maintenance of these two populations of cells was not dependant on circulating monocytes. Also, by generating CXCR4^CreERT2^; ROSA26^tdT^ mice and injecting tamoxifen to label all bone marrow haematopoietic stem cells (HSC), it was observed that PVMs were almost entirely tdT^−^, which illustrated that PVMs were not derived from HSC.^23^ In comparison, dural macrophages and choroid plexus macrophages partially come from the circulation.^17,24^

Therefore, BAMs can be divided into two groups based on their turnover and potential self-renewal ability. One group includes PVMs and leptomeningeal macrophages, which can self-renew and are not replenished much by peripheral cells. The other group comprises dural macrophages and choroid plexus macrophages, which allow for supplementation by circulating cells (Fig. 2). As for the effect of depletion using clodronate and other approaches that were later discovered, nearly all BAMs can be removed in the first place. However, dural and choroid plexus macrophages can be fully replenished by peripheral cells at a later stage, resulting in relatively specific PVM clearance.

PVMs are highly stable and are not often exchanged by peripheral monocytes physiologically.^1,25^ However, it is worth mentioning that in pathological states or when external interventions are made, PVMs allow for peripheral replenishment, especially through the infiltration of circulating monocytes or bone marrow transplantation.^26–28^ This phenomenon is attributed to the events of cell depletion (chemotherapy,^29^ specific ablation by drug or genetic strategies^30^), disruption of barrier (radiation^26^ or ageing^31^) or effects of stimulating factors (chitin administration^32^).

### Surface markers and anatomic distribution of perivascular macrophages

Several molecular players are associated with PVM identification, disease phenotypes and functions (Table 1). PVMs share myeloid cell markers such as CD45, CD11b, CX3CR1, CD64, etc. In particular, CD206 is a crucial marker to distinguish PVMs from microglia. During the development of brain-resident immune cells, CD206 becomes negative in microglia while remaining positive in BAMs, including PVMs.^17^

**Table 1:** Features of perivascular macrophages in the steady-state and diseased state

Condition	Species	Phenotype	Activation markers expressed	Gene expression tendency	Reference
**Physiological**					
	Mouse	CD45^hi^ CD11b^+^ Ly6C^low^ CX3CR1^low^ IBa-1^low^ F4/80^+^	MRC1^+^ CD163^+^ LYVE1^+^ MRC1^+^ PF4^+^ MHCII^hi^ CD80^+^ CD86^+^ CD40^+^	N/A	Goldmann *et al*.,^1^ Mrdjen *et al*.,^22^ Zeisel *et al*.^33^
	Human	CD45^low^ CD68^+^ CD11b^+^ CX3CR1^low^	MRC1^+^ CD163^+^ HLAII^+^ MRC1^+^ CD209^+^ CD40^+^ CD86^+^ CD80^−^		Fabriek *et al*.^34^
**Pathological**					
Neurocognitive alterations related to hypertension	Mouse	CX3CR1^low^ CD45^hi^ CD11b^+^ IBa-1^low^	MRC1^+^ LYVE1^+^	MRC1 ↓, LYVE1 ↓ PF4 ↓ CBR2 ↓ Ms4a7 ↔ CD74 ↑ CCL5 ↑	Faraco *et al*.^35^
Multiple sclerosis	Mouse	CD45^+^ CD68^+^	CD163^+^ MRC1^+^ HLAII^+^ CD209^+^ CD40^+^ CD86^+^ CD80^+^		Zhang *et al*.^36^
Experimental autoimmune encephalomyelitis	Mouse	CD68^+^	CD163^+^ MHCII^+^ CD40^+^ CD80^+^ CD86^+^ IL-1β^+^		Hofmann *et al*.^37^
Vascular disease	Mouse	CX3CR1^low^ CD45^hi^CD11b^+^ IBa-1^low^	MRC1^+^ CD163^+^ CD169^+^ CD36^+^		Gerganova *et al*.,^38^ Pedragosa *et al*.^39^
Alzheimer’s disease	Mouse	CX3CR1^low^ CD45^hi^CD11b^+^ IBa-1^low^	MRC1^+^ CD163^+^ SR-BI^+^ CD36^+^		Hawkes *et al*.,^32^ Park *et al*.^40^
SIV encephalitis	Macaque	ND	CD163^+^ Ki-67^+^	N/A	Nowlin *et al*.,^41^ Filipowicz *et al*.^42^
HIV encephalitis	Human	CD45^+^ CD14^+^	CD163^+^	N/A	Kim *et al*.^43^

N/A = not applicable; ND = not detected; SIV = simian immunodeficiency virus.

PVMs also have high expression levels of CD163, a haemoglobin-haptoglobin scavenger receptor that participates in a signal transduction cascade that produces cytokine.^44^ However, CD163 is also present in monocytes and inflammatory microglia, making it a less distinctive maker for the identification of PVMs.^34^ In addition, the lymphatic vessel endothelial hyaluronan receptor 1 (Lyve1), also known as the hyaluronan receptor, is expressed in lymphatic vessels and PVMs, but not in monocytes or microglia, making it more specific for labelling PVM than CD163 or CD206.^22,33^ Nonetheless, Lyve1 is not as sensitive as CD206 for the identification of PVMs.^45^

With the help of single-cell sequencing, it was recently discovered that CD11b^+^CD45^hi^CD206^+^ can isolate PVMs from CD11b^+^CD45^+^CD206^−^ microglia in flow cytometry.^1^ Moreover, another study claimed that BAMs were a separate population with a surface protein expression pattern of CD45^lo/+^CD11b^lo^F4/80^hi^CD64^hi^MeTK^+^CX3CR1^+^CD88^hi^.^22^ This population was positive for CD206 and CD38 while lacking the typical microglial expression of Siglec-H. Apart from that, Pf4, Cbr2 and Siglec1 were also identified as specific markers of BAMs.^45^ Using subclustering analysis, at least two distinct PVM populations characterized as Lyve1^+^ PVMs and MHC II^+^ PVMs were reported.^13^ Interestingly, Lyve1^+^ PVMs showed increased expression of anti-inflammatory (M2-like) polarization genes, such as *Ccr1*, *Cd163* and *Cd209a/f*, while MHCII^+^ PVMs exhibited proinflammatory (M1-like) genes, including *Cxcl9*, *Cxcl10* and *Cxcl13*. In terms of other subclusters of PVMs, a population of CX3CR1^−^CD45^low^Lyve1^+^ PVMs was identified that was capable of taking up macromolecules, which were located with CX3CR1^+^ PVMs.^23^ Additionally, the CX3CR1^−^Lyve1^+^ PVM population peaked at adulthood, comprising ∼20% of the Lyve1^+^ PVM population, and it declined in aged animals. Therefore, whether they have functions beyond classical CX3CR1^+^ PVMs remains to be determined.

Since the marker profiles of physiological status have been fully illustrated, it was also observed that in pathological conditions like neuroinflammation, most PVM-specific genes are downregulated, including *Mrc1*, *Lyve1*, *Cbr2* and *Pf4*.^20^*Ms4a7*, on the other hand, remained stably expressed, while antigen-presentation molecules (such as Cd74) showed a substantial increase.^20^ This could make the identification of diseased PVMs more difficult.

The distribution of PVMs may differ depending on the brain region, developmental stage or disease state. From an overall perspective, PVMs are located within the perivascular space, which is enclosed by a vascular basement membrane and the glial limitans. From a regional point of view, the first study to discover the distribution of PVMs at a brain-wide scale found that the hippocampus had the highest density of Lyve1^+^ PVMs.^46^ Furthermore, PVMs exhibit dynamic postnatal distribution, peaking between P10 and P20 depending on the brain region. The hippocampus, cortex and olfactory bulb have the most PVMs at the time point of P10, while the brainstem and cerebellum possess the highest number of PVMs at P20.^46^ Recent single-cell RNA sequencing studies indicated that PVMs tended to be in proximity to ICAM-1^+^ reactive postcapillary venule endothelial cells.^13^ In addition, most brain parenchymal vessels hosted Lyve1^+^MHCII^−^ PVMs with low-to-moderate CD45 expression.^23^ However, the underlying factors that contribute to PVM distribution characteristics varying in different physiological states remain unclear, and few studies have investigated the distribution of PVMs throughout the entire brain under diseased conditions.

Since the molecular properties of PVMs differ from those of microglia but are like those of other BAMs and peripheral monocytes, molecular markers (Fig. 2) and anatomic location are critical for the distinguishment of PVMs. Further studies using single-cell spatial transcriptomics are warranted to determine the location and migration of PVMs in various physiological and pathological conditions.

## Physiological functions of perivascular macrophages

### Phagocytosis

In the brain’s perivascular space, PVMs are poised to detect and phagocytose potentially hazardous foreign substances, such as bacteria and viruses, that can invade the parenchyma via the vasculature.^47,48^ Additionally, PVMs can engulf autogenous waste materials, such as cellular debris and amyloid-β protein.^32,49^ Studies have shown that PVMs can take up exogenous substances administered intraventricularly with the help of type I and II scavenge receptors^3,6^ and retain those macromolecules for an extended period.^25,50^

**Figure 3:**
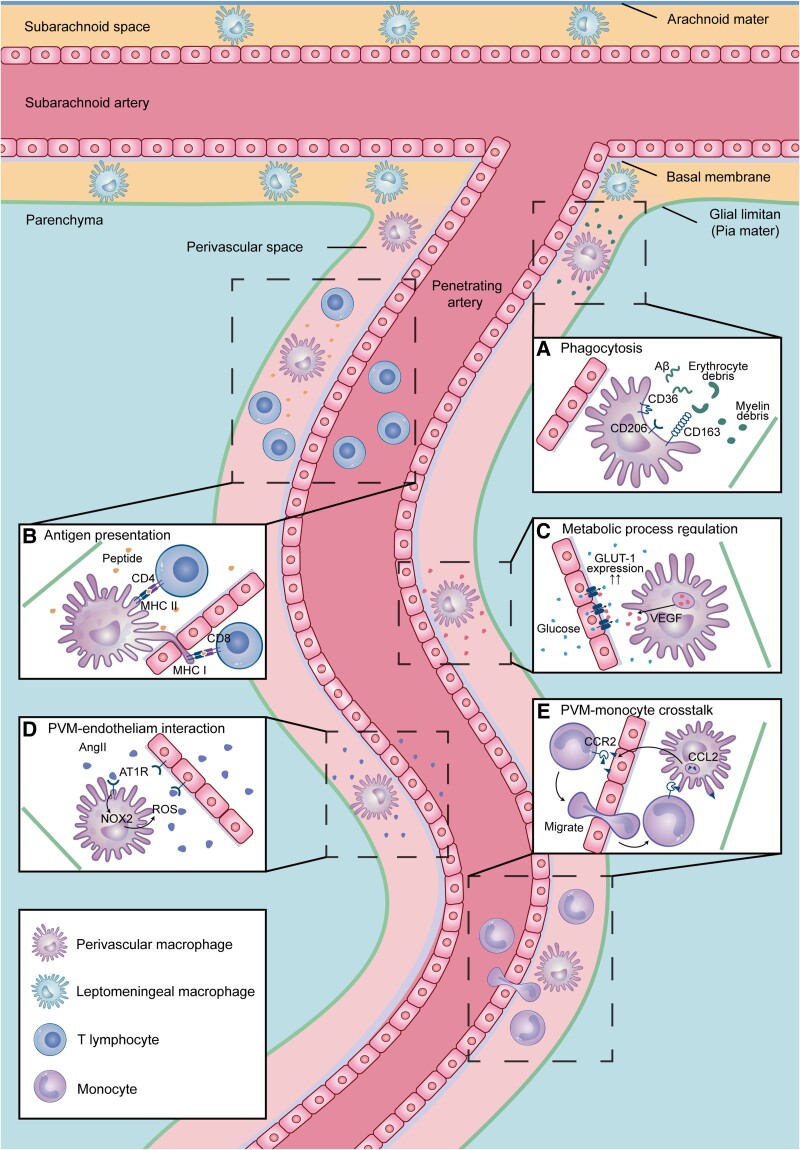
**Physiological functions and cellular interactions of PVMs**. (**A**) PVMs could phagocytize autogenous waste, including erythrocyte debris, myelin debris and amyloid-β protein. (**B**) PVMs interact with T cells via MHC I/MHC II and thus exert an antigen-presenting role. (**C**) Vascular endothelial growth factor (VEGF) produced by PVMs promotes the restoration of GLUT1 expression in endothelial cells under high-fat diet conditions. (**D**) Circulating Ang II activates AT1R in cerebral endothelial cells, promoting transcytosis and altering tight junctions, which allows peptides to reach the perivascular region to activate AT1R in PVMs under hypertensive conditions. The downstream Nox2 increase aggravates vascular oxidative stress, which in turn accentuates endothelial and PVM dysfunction and blood–brain barrier (BBB) leakage. (**E**) PVMs secrete CCL2, which subsequently anchors to the plasma membrane of endothelial cells and recruits monocytes to the brain through CCR2 on monocytes. PVM = perivascular macrophage.

A separate study revealed that the scavenging function of PVMs is attributed to the presence of CD206, a transmembrane glycoprotein involved in the uptake of glycoproteins and carbohydrate-containing structures,^51^ as well as the scavenger receptor CD163 (Fig. 3A).^52^ Moreover, bone marrow-derived monocytes have been shown to engraft into the PVS and demonstrate phagocytosis abilities after whole-body, radiation-induced PVM depletion.^53,54^ However, the underlying mechanism of monocyte-contributed PVM replenishment, as well as the potential functional distinction between infiltrated monocytes and resident PVMs, still requires further investigation.

### Antigen presentation

PVMs with metamorphic ability^55^ appear to function as antigen-presenting cells (APCs), as evidenced by major histocompatibility complex (MHC) molecule expression.^56^ The MHC class I receptor on PVMs is responsible for promoting CD8 T-cell infiltration into the brain during viral infection. A study that specifically deleted H2-Db of MHC class I from CNS-myeloid cells using CX3CR1^CreER^ mice, which includes PVMs, revealed a reduced number of infiltrating CD8 T cells in the brains of mice.^57^ This reduction occurred regardless of the proliferation and death rates of the CD8 T cells, indicating that H2-D^b^ MHC class I was crucial for antigen presentation and immune cell recruitment during Theiler’s murine encephalomyelitis virus infection. However, CX3CR1 is expressed in PVMs, microglia, monocytes^1^ and in infiltrated APCs, such as dendritic cells and peripheral-engraft macrophages under certain pathological conditions.^58^ Besides, both PVMs (CD206^+^) and dendritic cells (CD11c^+^LysM^+^) can activate CD8 T cells similarly but not identically.^59^ Therefore, it is difficult to determine the extent to which a particular cell type contributes the most to antigen presentation. Thus, in-depth research into how PVMs present antigens and whether this process requires assistance from other cell types is warranted (Fig. 3B).

### Maintenance of vascular integrity

The fenestrated microvasculature in the area postrema lacks tight connections and has a less compact blood–brain barrier (BBB).^60^ Researchers discovered that the PVMs in this location adsorbed 10- to 70-kDa dextran, a range similar in size to most serum proteins and blood-borne macromolecules from the vasculature.^61^ This prevented most of the vasculitic toxicities from entering the brain parenchyma, demonstrating that by engulfing those foreign substances, PVMs contributed to maintaining vascular integrity.

In the cochlear stria vascularis, perivascular-resident macrophage-like melanocytes (PVM/Ms) regulate intrastriatal fluid–blood barrier permeability by expressing pigment epithelium-derived factor, which further controls the expression of tight junction-associated proteins, including occludin, ZO-1, and vascular endothelial cadherin.^62^ In addition, PVM/M-conditioned media was found to significantly decrease endothelial monolayer permeability.^63^ Although PVM/Ms and PVMs have slightly different names, their distribution characteristics and functions are similar. However, whether PVMs regulate tight junctions of arteriole endothelial cells through a related mechanism in the brain parenchyma like PVM/Ms still needs to be clarified by *in vivo* experiments.

Recent studies have indicated that PVMs can be attracted by extracellular ATP and migrate to lesioned endothelial sites, extending filopodia or lamellipodia to physically adhere to both endothelial ends.^64^ Through adhesion, PVMs can generate mechanical force to pull the endothelial ends together and facilitate ligation, thereby repairing the vessel rupture. Moreover, CNS macrophages can moderate neurovascular development via CD95L.^65^ Since CD95 receptor activation on neurons and endothelial cells is necessary for proper synaptic activity and vascular remodelling, CD95L-expressed macrophages may orchestrate this CD95-dependent neurovascular reconstruction through SFK and PI3K-mediated actions.

However, in pathological conditions such as hypertension, PVMs were found to be indirectly involved in BBB disruption, an increase in transcytosis and a reduction in tight junction proteins through elevated oxidative stress.^66^

### Lymphatic drainage

The perivascular pathway, glymphatic system, meningeal lymphatic vessels, paravascular pathway and olfactory/cervical lymphatic drainage route are the five main pathways contributing to the clearance of waste and interstitial fluid (ISF) that have been found in the brain.^67–69^ Importantly, recent findings suggested that PVMs may actively participate in the facilitation of glymphatic-lymphatic drainage.

ISF and soluble substances are carried out from the brain parenchyma along perivascular pathways via the intramural periarterial space within a layer of vascular smooth muscles (VSMCs).^70,71^ PVMs aid in intramural peri-arterial clearance by absorbing particles in size ranging from 2 to 1 μm.^72^ Regarding VSMC tone, Lyve1^+^ PVMs have been shown to engage with hyaluronan on VSMCs and induce downstream activation of pericellular matrix metalloproteinase (MMP)-9, which prevents collagen deposition and arterial stiffness.^73^ Moreover, the activity of MMPs was found to be significantly reduced in the brains of PVM- and leptomeningeal macrophage-depleted mice. This suggests that macrophages might regulate extracellular matrix (ECM) degradation through the production or activation of MMPs, which ultimately affect arterial stiffness.^74^

The glymphatic system drains ISF and CSF from the para-arterial space through the glial parenchyma to the para-venous area, eventually entering the internal cerebral vein.^75^ The paravascular pathway is part of the glymphatic system, transporting fluid from the subarachnoid space to Virchow–Robin spaces in the same direction as the blood flow.^76^ The absence of PVMs would have a functional impact on the glymphatic system, as evidenced by the significant reduction that was observed in the diffusion rate to the parenchyma following ovalbumin injection through the cerebellar bulbar cisterna.^77^ In addition, the drainage of ovalbumin into deep cervical lymph nodes was also found to be decreased in PVM-depleted mice.

### Regulation of metabolic process

It was found that an acute high-fat diet reduced glucose transporter-1 (GLUT1) expression in endothelial cells, decreasing glucose absorption in the brain.^78^ However, the suppression of endothelial GLUT1 was temporary and levels were restorable with the help of myeloid cell vascular endothelial growth factor (VEGF) synthesis, including VEGF from PVMs. The same study also found that selective VEGF ablation in myeloid cells reduced the expression of endothelial GLUT1 and brain glucose uptake (Fig. 3C). However, a more selective depletion of VEGF in PVMs still needs to be examined. High-fat diets also increased inducible nitric oxide synthase (iNOS) expression in hypothalamic PVMs, which triggered neuroinflammation and vascular hyperpermeability but which could be alleviated by iNOS inhibition.^79^ Another study found that blood lipids were integrated into PVMs and accumulated in the cytoplasm as lipid burden increased with age.^80^ More research will be needed to comprehensively understand the role of PVMs in regulating metabolic homeostasis in the CNS.

### Interactions of perivascular macrophages with cells around the perivascular space

Diverse brain-resident cells such as PVMs, smooth muscle cells, microglia, pericytes and endothelial cells are located in or around PVS and may intimately interact with one another. Aside from these cells, leptomeningeal macrophages in the subarachnoid space are closely related to PVMs. Moreover, as the interface site between the brain parenchyma and the periphery, the PVS also contains infiltrating immune cells such as monocytes and lymphocytes.^31,81^ Hence, reciprocal interactions between PVMs and other cell types around the PVS may have profound effects on the maintenance of brain functions.

#### Brain-resident cells

##### Leptomeningeal macrophages

Leptomeningeal macrophages are a type of myeloid cell situated in the subarachnoid space. Whether the subarachnoid space connects to the perivascular space containing PVMs is currently a matter of debate.^67^ With the help of scanning and transmission electron microscopy, basal arteriolar perivascular spaces have been reported to connect directly with the subarachnoid space,^82,83^ while cortical periarteriolar and perivenular spaces communicate with the subpial space rather than the subarachnoid space.^67,84^ PVM and leptomeningeal macrophages are both phagocytic cells produced in response to macromolecules such as dyes, bacteria and erythrocytes.^47,49,50^ However, the scavenging roles of and interactions between these two cell types remain unclear.

As previously mentioned, leptomeningeal macrophages and PVMs share a common origin. Research has shown that perinatal leptomeningeal macrophages located in the subarachnoid area had the ability to migrate to perivascular space and transform into PVMs in an integrin-dependent manner postnatally.^9^ Following that case, it was found that the pool of PVMs could be replenished through either self-proliferation or subsequent leptomeningeal macrophage infiltration during the early postnatal period. This finding aligns with the observation that PVMs and leptomeningeal macrophages exhibit similar protein expression markers such as Lyve1 and MHC II.^22^

However, it is unknown whether leptomeningeal macrophages continue to migrate to the PVS and transform into PVMs in adulthood. And, if migration does occur, the underlying mechanism and signal that trigger this event to occur remain unknown. Recently, it was found that adult mouse meninges contained HSCs that were actively dividing, suggesting that these meningeal HSCs could replenish dural macrophages.^85^ Nevertheless, whether these HSCs can differentiate into PVMs and leptomeningeal macrophages requires further investigation.

##### Vessel smooth muscle cells

Vessel smooth muscle cells (SMCs) are an integral component of neurovascular units and are essential in regulating cerebral blood flow.^86^ As for their communication with PVMs, Masuda *et al*.^9^ found that the establishment of PVMs requires the presence of arterial VSMCs. Through experiments using *Notch3*^−/−^ mice, they observed a substantial decrease in VSMCs in arteries and arterioles, which was accompanied by a reduction in the number of PVMs. By generating SMMHC^CreERT2^; Rbpj^flox/flox^ mice to specifically knock out the *Rbpj* gene (a major downstream transcription factor of the Notch pathway) in SMCs, researchers still detected a reduction in PVMs, indicating that the presence of arterial SMCs is crucial for the proper distribution of PVMs during development.

Drieu *et al*.^74^ discovered that the depletion of parenchymal border macrophages (PBM), which contain PVMs and leptomeningeal macrophages, impaired arterial motion. This finding indicated a potential interaction between PVMs and VSMCs in regulating vasodilation and contraction. Using scRNA-seq and bioinformatics analysis, they identified five clusters within the PBM population. Among these clusters, one showed elevated expressions of scavenger markers such as Lyve1 and CD163 and further demonstrated specific interactions with VSMCs within arteries. However, the underlying ligand-receptor pairs were not elucidated in this study.

As mentioned previously, Lyve1^+^ PVMs can induce MMP9-dependent collagen degradation surrounding VSMCs in mice and prevent arterial sclerosis.^73^ Accordingly, studies found that collagen type IV and laminin, two ECM proteins, were accumulated near VSMCs in PBM-depleted mice.^74^ Furthermore, MMP activity was observed to be significantly reduced in the brains of PBM-depleted mice, which provides additional evidence supporting the notion that macrophages may regulate ECM degradation through the production of MMPs, thus influencing arterial stiffness.

##### Microglia

Microglia and PVMs possess phagocytosis properties to a certain extent and are vital for maintaining brain homeostasis after birth.^87^ Microglia can take up parenchymal ISF amyloid-β, whereas PVMs can degrade perivascular amyloid-β.^88^ When it comes to the crosstalk between microglia and PVMs, recent evidence in a mouse model of Alzheimer’s disease indicated that the expression of secreted phosphoprotein 1 (SPP1/osteopontin) was upregulated in PVMs, which strengthened the phagocytic function of microglia, resulting in synaptic loss.^89^

Peripheral-derived macrophages such as HSC-derived macrophages and monocyte-derived macrophages can engraft into microglia niches.^30,90,91^ However, whether brain-resident PVMs can also engraft into microglia niches remains unknown. Recently, studies using time-lapse imaging in zebrafish discovered that a group of mrc1a^+^ (known as CD206^+^ in mice) microglial precursors seeded the brain before traditional microglia appeared.^92^ These early microglial precursors were independent of PU1^+^ yolk sac-derived microglia but were located in the lymphatic vasculature around the brain, suggesting a potential replenishment of microglia by PVMs. It was also discovered that at E12.5 in mice, intraventricular CD206^+^ macrophages, which were supplied from the roof plate of the brain, frequently infiltrated the pallium and subsequently differentiated into CD206^−^ microglia.^93^ However, the studies mentioned above primarily focused on the transformation of CD206^+^ cells into microglia during the developmental stage. It is still unclear whether similar transformations can occur in adulthood or under certain pathological conditions.

##### Pericytes

Pericytes are perivascular cells embedded within the capillary endothelium basement membrane and are regarded as crucial components of BBB.^94,95^ In a mouse experimental allergic encephalomyelitis model, a family of ECM proteins called chondroitin sulphate proteoglycans (CSPGs) was highly abundant within inflamed perivascular cuffs.^96^ These CSPGs produced proinflammatory cytokines that stimulated pericytes. Then, those pericytes enhanced the infiltration of leucocytes, particularly monocyte-derived macrophages, into the PVS of the brain, suggesting that pericytes may contribute to PVM turnover.

Additionally, in the ischaemic brain, the SMA^low/undetectable^ pericytes proliferated substantially during the subacute phase after injury and could differentiate into macrophage-like cells.^97^ Flow cytometry analysis of Ai14;PDGFRβ^Cre^ and Ai14;PDGFRβ^CreERT2^ ischaemic stroke mice revealed that 12–18% of macrophages were PDGFRβ positive (showing positive fluorescence of tdT). The underlying regulatory mechanisms and functions of the SMA^low/undetectable^ pericyte-derived macrophage-like cells await further exploration.

Regarding the potential interaction between pericytes and PVMs, it was found that pericyte-conditioned medium promoted the phagocytic activity of macrophages by enhancing both signal transducer and activator of transcription 3 (STAT3) phosphorylation and scavenger receptor expression.^98^ Additionally, that research also revealed that macrophages can produce trophic factors to facilitate PDGFRβ signalling in pericytes, thereby synergistically benefiting post-stroke tissue repair and functional recovery. However, those cell experiments failed to fully reflect the *in vivo* condition. As such, future *in situ* studies are warranted to accurately investigate the interaction between the two.

##### Endothelial cells

Owing to the proximal location of endothelial cells and PVMs, these two cell types may interact with one another to some extent. Indeed, in the hypertension model, circulating Ang II was shown to activate AT1R expressed in cerebral endothelial cells, modifying tight junctions and increasing transcytosis, which facilitated the entrance of blood-borne peptides into the PVS and activated AT1R in PVM.^66^ The downstream NADPH oxidase 2 (Nox2) elevation further exacerbated vascular oxidative stress, bringing about endothelial and PVM dysfunction and BBB leakage (Fig. 3D). Additional research showed that although endothelial cells and PVMs can activate the hypothalamic-pituitary-adrenal (HPA) axis under the stimulation of IL-1 or lipopolysaccharide (LPS), PVMs can produce factors to restrain the endothelium-inflamed condition.^99^ The paracrine mediators of bidirectional interactions between these two cell types have yet to be identified.

#### Circulating cells

##### Monocytes

PVMs were previously thought to be derived from circulating monocytes.^4^ However, scientists later found that PVMs originated from perinatal leptomeningeal macrophages after birth.^9^ During the physiological state, the turnover of PVMs is low, which means that peripheral-derived monocytes rarely access the perivascular space and transform into PVMs.^1,25,32^ However, in pathological conditions, circulating monocytes were found to infiltrate into the PVS and participate in inflammatory responses.^31,100^

A recent study using Flt3 promoter to label cells of definitive haematopoietic origin showed that compared with wild-type mice, APP/PS1 mice had a greater degree of monocyte infiltration in the interface areas (perivascular space, choroid plexus and meninges).^101^ Additionally, monocytes were recruited to amyloid plaques. The migration of monocytes was also supported by an experiment using CCR2^RFP/+^ mice, which showed that peripherally originated RFP^+^ monocytes can be detected in the perivascular space (Fig. 3E).

##### Lymphocytes

PVMs are strategically positioned within the perivascular space, a structure that lymphocytes must cross to reach the brain parenchyma. PVMs have been shown to function as APCs through MHC class I, which could facilitate CD8 T-cell infiltration into the brain parenchyma.^57^ Additionally, PVMs may extend their cellular processes across the vessel wall to the vascular lumen and present antigens to cells in the blood, including T lymphocytes.^55^ PVMs have also been discovered to form an intravascular niche for CD8 T-cell localization.^102^ Through two-photon intravital microscopy (TP-IVM) brain imaging, it was witnessed that in the early stages of disease, CD8^+^ T cells crawled along the inner vessel wall towards PVMs that were located on the abluminal side of large post-capillary venules. It was suggested that the interaction between PVM and CD8^+^ T cells across the BBB may initiate an inflammatory cascade, leading to BBB alteration and neuropathology.

In a mouse model of degenerative synucleinopathy, ablation of PVMs by clodronate promoted the expression of vascular cell adhesion protein 1 (VCAM-1), an adhesion molecule required for cell trafficking across the BBB.^77^ Additionally, in that model, the depletion of PVMs enormously increased CD4 and CD8 extravasation, indicating that PVMs might act as gatekeepers to control the trafficking of lymphocytes into the CNS.

Considering that B lymphocytes also infiltrate the brain during disease development,^103^ there is a possibility of interaction between the B lymphocytes and PVMs. However, research in this area is limited. Attention to the crosstalk between PVMs and B lymphocytes is warranted for future studies.

## Perivascular macrophages in the diseased CNS

As potential gatekeepers of the CNS, PVMs are hypothesized to regulate immune cell trafficking from the blood and CSF into the brain parenchyma, as well as to facilitate the exchange of various molecules between the blood and the CNS. Therefore, PVMs may also play an essential role in the pathogenesis of various brain disorders (Table 2).

**Table 2:** Perivascular macrophages in distinct disease models

Disease	Model	Brain region	PVM manipulation	Function of PVM	Reference
**Neurodegenerative disease**					
CAA	TgCRND8 transgenic mice	Cortex and hippocampus	CLO, chitin	Promote Aβ clearance	Hawkes *et al*.^32^
CAA	J20 transgenic mice	Neocortex and basal ganglia	SR-B1^+/+^, SR-B1^−/−^, SR-B1^+/−^	Facilitate Aβ clearance through SR-B1, improve neurocognitive function	Thanopoulou *et al*.^104^
Alzheimer’s disease	Aβ regional perfusion, Aβ i.v. administration, Tg2576 transgenic mice	Somatosensory cortex	CLO, bone marrow chimeras	Mediate neurovascular dysfunction via CD36 and NOX2	Park *et al*.^40^
Alzheimer’s disease	APPswe/ps1 mice, APP23 mice	Cortex and hippocampus	CCR2^−/−^ and CCR2^+/+^, bone marrow chimeras	Clear Aβ in a CCR2-dependent manner	Mildner *et al*.^26^
Parkinson’s disease	Transgenic mice overexpressing α-synuclein	N/A	N/A	Promote clearance of toxic α-synuclein	Kovacs *et al*.^105^
**Cerebrovascular diseases**					
Hypertension	Ang II administration, BPH mice	Cortex and hippocampus	CLO, bone marrow chimeras	Produce ROS, cause neurovascular and cognitive dysfunction	Faraco *et al*.^35^
Ischaemic stroke	tMCAO	Cortical and subcortical regions	CLO	Recruit granulocyte, promote VEGF expression, increase BBB permeability, and cause neurological dysfunction	Rajan *et al*.,^106^ Pedragosa *et al*.^39^
Ischaemic stroke	Mice exposed to alcohol chronically	Cortical and subcortical regions	CLO	Key players on alcohol-induced aggravation effect of ischaemic lesions	Drieu *et al*.^107^
Cerebral haemorrhage	Experimental subarachnoid haemorrhage	Cortical and subcortical regions, subarachnoid space and ventricular system	CLO	Contribute to neuronal cell death and perivascular inflammation	Wan *et al*.^49^
Cerebral haemorrhage	Experimental intracerebral haemorrhage	Right striatum	Bexarotene administration	Phagocytose erythrocyte via Axl and CD36	Chang *et al*.^108^
High-fat diet	Mice with a high-fat diet feeding	Hypothalamus, motor and somatosensory cortex, nucleus accumbens	VEGFa^flox/flox^ LysMCre^+/−^	Increase the expression of VEGF and GLUT1, sustain cerebral glucose absorption and reduce cognitive impairment.	Jais *et al*.^78^
**Inflammatory condition**					
Bacterial meningitis	Wistar rats received Streptococcus pneumonia intracisternal injection	N/A	CLO	Protective, facilitates leucocyte infiltration	Polfliet *et al*.^47^
Viral encephalitis	Breeding pairs of macrophage fas-induced apoptosis mice, intranasal injection of VSV	N/A	CLO	Detrimental facilitates leucocyte infiltration	Steel *et al*.^109^
**Autoimmune disease**					
Multiple sclerosis	Lewis rats injected with MOG	N/A	CLO	Accelerate the development of clinical symptoms	Polfliet *et al*.^110^
Multiple sclerosis	Lewis rats injected with MOG	Parietal cortex and hippocampus	N/A	Allowed the T lymphocyte extravasation and CNS-parenchymal infiltration by antigen presentation	Walther *et al*.^111^

Aβ = amyloid-β; BBB = blood–brain barrier; CAA = cerebral amyloid angiopathy; CLO = clodronate; i.v. = intravenous; MOG = myelin oligodendrocyte glycoprotein; N/A = not applicable; ROS = reactive oxygen species; tMCAO = transient middle cerebral artery occlusion; VSV = vesicular stomatitis virus.

### Neurodegenerative diseases

#### Alzheimer’s disease

Alzheimer’s disease is characterized by the abnormal accumulation of amyloid-β deposits and neuronal tau tangles.^112^ Regarding amyloid-β clearance, the main pathways include degradation, the BBB, ISF bulk flow and CSF absorption.^88^ The phagocytosis of amyloid-β by PVMs is considered a protective function and thus far received the most attention in research.

A recent study showed that ablation of PVMs with clodronate liposomes in 4-month-old TgCRND8 mice worsened cerebral amyloid vasculopathy.^32^ It is important to note that clodronate liposomes, which are ingested and digested by macrophages, are not specific to the depletion of PVMs but are also involved in the depletion of other macrophages.^113^ As clodronate concentrates in the cytoplasm of cells, it disrupts cellular metabolism and leads to apoptosis.^114^ However, this drug does not have an impact on microglia or astrocytes.^113^

To identify the receptors in PVMs corresponding to the regulation of amyloid-β deposition, Thanopoulou *et al*.^104^ found that scavenger receptor class B type I (SR-BI), an HDL receptor expressed in PVMs, was elevated in the brains of mice with Alzheimer’s disease. SR-BI reduction accelerated cerebrovascular and parenchymal amyloid plaque deposition in the cortex and hippocampus, implying that PVM-derived SR-BI played a role in amyloid-β clearance.

To specifically target PVMs, brain-shielding bone marrow chimeric mice were generated, which only allowed for the exchange of PVMs without mononuclear phagocytes entering into the brain parenchyma. After the transplantation of *CCR2*^−/−^ bone marrow cells into APP^swe^ mice, the amyloid burden in the brain was significantly increased.^26^ Thus, it was concluded that PVMs modulated amyloid-β deposition by clearing them using a CCR2-dependent mechanism. Moreover, recent findings suggested that PVMs play a role in regulating CSF flow dynamics. By intracisternal injection of macrophage colony-stimulating factor (M-CSF), the MMP activity of PVMs was boosted, which resulted in a short-term improvement in CSF flow.^74^ This made PVMs a promising target for pharmacological interventions to alleviate brain clearance deficits associated with ageing and Alzheimer’s disease.

Concerning the pathological effect of PVMs, one previous study noted that by activating the scavenger receptor CD36 in PVMs, these cells became the source of reactive oxygen species responsible for the cerebrovascular effects of amyloid-β.^115^ By first clearing PVMs using an intracerebroventricular injection of clodronate and then transplanting PVMs originating from *CD36*^−/−^ or *Nox2*^−/−^ bone marrow chimeras, it was found that deletion of CD36 or Nox2 from PVMs abrogated the deleterious vascular effects of amyloid-β.^40^ Moreover, PVMs could induce microglia to engulf synapses and cause synaptic loss in mouse models of Alzheimer’s disease by producing SPP1.^89^

Overall, PVMs may play a dual role in Alzheimer’s disease, as they can either clear amyloid-β from the brain through the CSF flow or produce pro-inflammatory cytokines that damage the neurovascular unit. Future studies should identify the critical factors determining when PVM function shifts from protective to harmful in brains affected by Alzheimer’s disease. Moreover, the impact of disease progression on the function of PVMs should be further investigated.

#### Parkinson’s disease

The presence of eosinophilic inclusion bodies (Lewy bodies) in the cytoplasm of the remaining neurons in the substantia nigra is one of the most prominent pathological characteristics of Parkinson’s disease.^116^ In degenerative synucleinopathy mice that were part of a mouse model of Parkinson’s disease, the number of PVMs was significantly increased on the injured side of the brain, which was mainly derived from the local proliferation of PVMs.^77^ After the depletion of PVMs, the infiltration of lymphocytes was exacerbated in the lesion site, and the spreading of Lewy-like pathology was profoundly aggravated, which indicated that the increase in PVMs may have served a protective role. Further study also detected that PVMs can take up and facilitate the clearance of toxic α-synuclein protein aggregates.^77,105,117^ Additionally, obstructing meningeal lymphatic drainage via ligating deep cervical lymph nodes exacerbated the accumulation of α-synuclein in the perivascular space,^118^ suggesting a functional role for PVMs and lymphatic drainage in α-synuclein clearance.

### Cerebrovascular diseases

Cerebrovascular diseases are those that affect blood flow in the brain.^119^ Hypertension is a risk factor for cerebrovascular diseases, as it is known to be associated with an increased number of macrophages and monocytes in the walls of cerebral blood vessels.^120^ In fact, PVMs can sense and respond to environmental signals and are critical in the homeostasis and remodelling of arterial functions.^121^ For example, they can be activated via AT1R and produce reactive oxygen species (ROS) via Nox2, causing vascular oxidative stress and exacerbating hypertension-related neurovascular uncoupling.^35,66^ Pharmacological depletion of microglia and PVMs by CSF1R inhibitor (PLX5622) also prevented short-term memory impairment in Ang II-induced hypertensive mice.^11^ Since CSF1R inhibitors deplete both brain macrophages and microglia, it is not possible to distinguish which cell type played a more major role. Therefore, a method to ablate the specific cell types should be adopted.

High-fat diet feeding, another risk factor for cerebrovascular disease, was reported to increase both the number of and VEGF immunoreactivity in perivascular CD206-positive macrophages.^78^ The VEGF synthesized by PVMs could help rescue GLUT1 expression in endothelial cells and enhance glucose absorption in the brain. This was further confirmed by knocking out VEGF in myeloid cells using VEGFa^flox/flox^; LysM^Cre+/−^ transgenic mice. The expression of endothelial GLUT1 was reduced and brain glucose uptake and cognitive function were impaired. It is worth noting that LysM^Cre+/−^ mice may express Cre recombinase not limited to PVMs and a more specific method to deplete VEGF in PVMs should be considered in future studies.

PVMs are also involved in cerebral ischaemia, a common type of cerebrovascular disease.^38^ One study of a middle cerebral artery occlusion (MCAO) rat model demonstrated that the number of CD206^+^ macrophages increased significantly both at the leptomeninges (leptomeningeal macrophages) and along the vessels penetrating the cortex (PVMs).^106,122^ Another study reported that the population of CD163^+^ brain PVMs was maintained, while their gene expression profile changed dramatically.^39^ Additionally, CD163^+^ brain PVMs could secrete VEGF, which led to increased vascular permeability and leucocyte migration into the brain. Whether the infiltrated leucocytes were responsible for downstream cerebral blood flow hypoperfusion and neurological dysfunction needs further examination.^39,106^

Although depletion of PVMs before disease onset reduced leucocyte recruitment and prevented leakage of pial and cortical vessels, no significant changes occurred in infarct volume or neurological function.^39^ This may be related in part to the intervention timing and other undetermined factors. In a study focusing on the relationship between alcohol and ischaemic stroke, an elevated number of PVMs was found in alcohol-exposed mice, which was related to exacerbated inflammatory responses following a secondary insult, such as LPS challenge or ischaemic stroke.^107^ Furthermore, the deleterious effect of alcohol exposure could be alleviated by the depletion of PVMs using clodronate liposomes.

In cerebral haemorrhage disease, PVMs play crucial roles as well. For example, a study of experimental subarachnoid haemorrhage found that PVM depletion post-subarachnoid haemorrhage improved neurological scoring and reduced neuronal cell death and perivascular inflammation, whereas PVM depletion pre-subarachnoid haemorrhage only reduced perivascular inflammation.^49^

### Inflammatory conditions

The physical structure of the BBB effectively protects the CNS from pathogens such as viruses and bacteria that may enter the brain.^94^ It has been suggested that PVMs, rather than microglia, are the primary cells infected productively by simian immunodeficiency virus (SIV).^48^ One study showed that CD163^+^ macrophages accumulated in the PVS of SIV encephalitis mice and played a role in recruiting peripheral monocytes.^41^ In another study, a significant increase in CD163^+^Ki-67^+^ cells was observed in SIV-infected encephalitis mice compared to both uninfected and SIV-infected animals without encephalitis.^42^ Additionally, the expression of CSF1R in the SIV-infected PVMs was found to be markedly increased, suggesting the possibility of developing a targeted approach for infected brain macrophages.^123^

In an experimental pneumococcal meningitis model, the depletion of meningeal macrophages and PVMs led to severe sickness, accompanied by increased bacterial counts in both blood and CSF.^47^ This indicated that PVMs may protect against bacterial infection. However, in acute encephalitis induced by the vesicular stomatitis virus (VSV), selective depletion of PVMs suppressed the granulocytic response and lymphocyte accumulation, resulting in the suppression of encephalitis.^109^ This serves as a reminder that PVMs may have a detrimental function in viral infection. Reasons for the apparent opposite functions of PVMs in bacterial versus viral infections deserve further investigation.

In addition to studying infection through an adult animal model, scientists turned to an infant model to find out if the distribution of myeloid cells affects disease. It was discovered that in neonatal macaques, the number of CD206^+^ PVMs in uninfected newborns was substantially higher than in uninfected adults.^124^ Moreover, a subset of unique CD206^+^CD163^−^ PVMs was found in newborns that underwent programmed cell death after SIV infection, which limited the size of the virus reservoir in infants’ brains. This study suggests that these subsets of PVMs can protect infants from SIV infection.

Overall, PVM can engulf and remove pathogens, or act as a hiding place for them. To that end, more research should be performed to improve the efficiency of PVM phagocytosis and pathogen removal.

### Autoimmune disease

Multiple sclerosis is a chronic autoimmune demyelinating disease that primarily affects the CNS of young adults. Its key pathological features are demyelinating lesions linked to perivascular leucocyte infiltration, astrogliosis and axonal damage.^125^ Notably, PVMs have been found to possess dual roles in multiple sclerosis. Protective functions are reflected in phagocytosis and degradation of the myelin antigen, while harmful functions are present through antigen presentation and facilitation of T-cell infiltration.^36^

One study demonstrated that the expression of ED1 (also known as CD68, a phagocytosis function marker) in PVMs increased during the acute and chronic relapsing stages of experimental allergic encephalomyelitis.^126^ The ED1-positive macrophages were considered aggravating relapse factors and the underlying mechanisms were investigated. PVMs were found to facilitate T lymphocyte extravasation and parenchymal infiltration by antigen presentation.^111^ The T lymphocyte extravasation effect may be mediated by the expression of VCAM-1, ICAM-1 and the chemokines MCP-1 and MIP-1α.^37^ Depletion of the PVMs suppressed clinical symptoms and weight loss in an animal model of multiple sclerosis.^110^ Further studies should be done to evaluate the precise effects of specific PVM manipulation in this autoimmune disease.

Overall, since most studies have not distinguished PVMs from infiltrating peripheral monocyte-derived macrophages, the exact contribution of PVMs to immune homeostasis in multiple sclerosis pathophysiology is unclear. This is mainly attributed to a lack of identification methods. Nevertheless, the development of new research strategies may help increase cell type-specific manipulation of functions. For example, parabiosis of wild-type mice and GFP mice allowed researchers to observe the infiltration of peripheral GFP^+^ cells into a GFP-negative recipient.^127^ CXCR4^Cre^ mice could also be used to selectively label peripheral bone marrow-derived cells.^128^

### Traumatic brain injury

Traumatic brain injury (TBI) is an acquired insult to the brain caused by an external mechanical force that can result in temporary or permanent impairment.^129^ Research has shown that CD14 is required for an immune response after TBI.^130^ It was also observed that following TBI, CD14^+^ cells observably increased near the lesion site and neighbouring perilesional areas. Notably, CD14 was detected to be constitutively expressed in PVMs rather than in parenchymal microglial cells, which indicated that PVMs may play a dominant role in TBI. Besides, IL-16 cell clusters were found in perivascular space in sites of initial spinal cord injury (SCI).^131^ Ultimately, the sirtuin 1 (SIRT1) agonist, SRT1720, could significantly promote functional recovery in SCI by lowering proinflammatory cytokine levels and PVM accumulation.^132^ However, the study did not reveal the exact function of PVMs in the pathogenesis of SCI.

### Epilepsy

Increasing evidence suggests a key role for neuroinflammation in epilepsy.^133^ After status epilepticus, PVMs and microglia expressed chemokine ligand 2 (CCL2) and recruited circulating monocytes expressing CCR2.^12^ Another study also confirmed persistent PVM activation in the hippocampus of epileptogenic humans and rats.^134^ The expression of CD68, CCL2 and the PVM marker CD163 correlated with the onset of the first insult and the incidence of spontaneous seizures, suggesting that these proteins contribute to epileptogenesis and epilepsy progression. Moreover, in chronic epileptic rats, the number of CD163^+^ PVMs was also positively correlated to BBB dysfunction.^134^

Therefore, PVMs may play a role in the inflammatory response before and after the onset of epilepsy. Whether PVMs can be precisely regulated to modulate the pathological processes in status epilepticus needs further investigation.

## Possible strategies targeting perivascular macrophages for CNS disease therapy

### Weakening damaging influences of perivascular macrophages

It has been reported that treatment with macrolide derivatives can reduce the production of IL-12 and alleviate macrophage neurotoxicity.^135^ In glioma models, using a CSF1R inhibitor before radiotherapy to eliminate macrophages contributed to better survival benefits.^136^ Besides, in encephalitis, selective activation of CSF1R signalling was found in infected brain macrophages, indicating prospects for creating a method that targets disease-associated brain macrophages without affecting resting, uninfected PVMs that display no CSF1R activation.^123^ More studies should also focus on other injurious receptors, such as AT1R, to weaken the detrimental effects of PVMs.

Furthermore, manipulating the metabolism of macrophages might be promising for reversing cognitive decline in ageing. Studies have demonstrated that in ageing macrophages, glucose metabolism was disturbed in response to a signal by PGE2.^137^ Specifically, PGE2 signalling via its EP2 receptor promoted glucose sequestration into glycogen, limiting glucose flux and mitochondrial respiration, ultimately leading to aggravated cognitive dysfunction. However, as previously mentioned, inhibiting the myeloid EP2 signal can rejuvenate cellular metabolism, inflammatory states, hippocampal synaptic plasticity and spatial memory.

### Enhancement of the protective function of perivascular macrophages

As mentioned above, PVMs have protective functions in various neurological disorders. For example, in intracerebral haemorrhage models, bexarotene administration increased the expression of phagocytosis receptors such as CD36 and Axl in macrophages,^108^ which was found to further increase erythrophagocytosis, reduce haematoma volume and improve neurological recovery. Subsequent studies could investigate whether the protective functions of PVMs can be promoted by physically or chemically modifying the protein expressions associated with phagocytosis, such as CD206, CD163, SR-B1, CCR2 and CD68, among others. Moreover, it is worth exploring whether manipulating PVM metabolism could facilitate these protective effects. Beyond that, PVM transplantation at an appropriate time during disease progression could also be a potential strategy to pursue.

## Future directions and open questions

Brain PVMs have attracted increasing attention for their various roles in supporting brain structure and maintaining physiological functions. They are also widely involved in degenerative, vascular, inflammatory and autoimmune complications and related diseases in either beneficial or deleterious manner depending on the circumstance. A deeper understanding of the phenotypic changes and regulatory signals of PVMs in different pathological conditions could pave the way for developing novel therapeutic strategies for treating brain disease. However, many unknown and critical issues still need to be addressed in the field of PVM research.

1. The specific cell surface markers (important for PVM identification), regulatory genes and underlying mechanisms required for understanding the cell fate of PVMs in different disease contexts and developmental stages remain largely unknown. The widely recognized PVM markers are CD206 (encoded by the *Mrc1* gene), Lyve1, CD163 and Pf4. However, they are more or less also expressed by cells other than PVMs. Using multilabel fluorescence-activated cell sorting, laser-capture microdissection and patch clamps accompanied by single-cell spatial transcriptome sequencing will help identify PVM markers and create specific mice targeting PVM based on those markers.
2. Currently, a less specific PVM-targeting strategy can be used to manipulate PVMs precisely. Clodronate administration and bone marrow transplantation are the most commonly used approaches for regulating PVMs. However, they fail to specifically target PVM. The specific type of transgenic mice available for PVM genetic manipulation nowadays is the Mrc1^CreERT2^ line. Still, it inevitably targets other BAMs and peripheral tissue-resident macrophages in addition to PVMs.^9^ Recently, a binary cre transgenic approach generating CX3CR1^Cre^:Lyve1^Cre^ mice was created to differentiate PVMs from microglia and to specifically target a subset of PVMs, which provided an innovative strategy for in-depth exploration of PVMs.^45^ Beyond that, a cre system using a transcription factor called c-MAF was also established, which found that conditional deletion of c-MAF in macrophage lineages using Lyve1^Cre^, LySM^Cre^, Csf1r^Cre^ and Maf^flox^ could cause PVM ablation in the brain and altered muscularis macrophage programme in the intestine.^138^ Therefore, developing strategies for targeting PVMs is strongly recommended for future PVM-associated treatments. Future studies using PVM-specific promoters or transcription factors are needed to target and explore PVM-specific functions and pathological roles.
3. At present, the profound mechanisms underlying the roles of PVMs in both physiological and pathological states remain inadequately studied. The use of PVM-specific transgenic mouse lines in combination with molecular profiling of cellular changes should help advance the understanding of PVM functions. For example, by crossing Mrc1^CreERT2^ mice with Ai9, tdT^+^-labelled PVMs and leptomeningeal macrophages can be induced by injecting tamoxifen. Those tdT^+^ cells are ideal for living images and flow cytometry analysis to reveal the proportion of PVMs in different states as well as changes in their expression profiles. Additionally, flow cytometry sorting of those cells can provide samples for single cell sequencing or cell culture study. By crossing Mrc1^CreERT2^ mice with iDTR or flox mice, it allows for specific ablation of PVMs or knocking out of PVM-expressing genes for functional study of PVM-mediated biological effects.
4. Research findings of PVMs from animal models do not fully reflect the alterations in human patients. Future studies using post-mortem brain slices would be of great value in facilitating the characterization of human PVMs in different diseases and brain regions.

Collectively, further research is warranted to learn more about PVMs, the important innate immune cells of the brain.
